# Environmental DNA in subterranean biology: range extension and taxonomic implications for *Proteus*

**DOI:** 10.1038/srep45054

**Published:** 2017-03-27

**Authors:** Špela Gorički, David Stanković, Aleš Snoj, Matjaž Kuntner, William R. Jeffery, Peter Trontelj, Miloš Pavićević, Zlatko Grizelj, Magdalena Năpăruş-Aljančič, Gregor Aljančič

**Affiliations:** 1 Society for Cave Biology, Tular Cave Laboratory, Oldhamska cesta 8a, 4000 Kranj, Slovenia; 2 Department of Animal Sciences, Biotechnical Faculty, University of Ljubljana, Groblje 3, 1230 Domžale, Slovenia; 3 Department of Life Sciences, University of Trieste, Via Licio Giorgieri 5, Trieste 34127, Italy; 4 Institute of Biology, Scientific Research Centre, Slovenian Academy of Sciences and Arts, Novi trg 2, 1000 Ljubljana, Slovenia; 5 Department of Biology, University of Maryland, College Park, MD 20742, USA; 6 Department of Biology, Biotechnical Faculty, University of Ljubljana, Večna pot 111, 1000 Ljubljana, Slovenia; 7 Biospeleological Society of Montenegro, Cara Lazarja 22, 81000 Podgorica, Montenegro; 8 Scientific Research Society Versus, Vitina bb, 88326 Vitina, Bosnia and Herzegovina; 9 University of Bucharest Research Institute, ICUB, Transdisciplinary Research Centre Landscape - Territory - Information Systems, CeLTIS, 91-93, Splaiul Independentei, 050095 Bucharest, Romania

## Abstract

Europe’s obligate cave-dwelling amphibian *Proteus anguinus* inhabits subterranean waters of the north-western Balkan Peninsula. Because only fragments of its habitat are accessible to humans, this endangered salamander’s exact distribution has been difficult to establish. Here we introduce a quantitative real time polymerase chain reaction-based environmental DNA (eDNA) approach to detect the presence of *Proteus* using water samples collected from karst springs, wells or caves. In a survey conducted along the southern limit of its known range, we established a likely presence of *Proteus* at seven new sites, extending its range to Montenegro. Next, using specific molecular probes to discriminate the rare black morph of *Proteus* from the closely related white morph, we detected its eDNA at five new sites, thus more than doubling the known number of sites. In one of these we found both black and white *Proteus* eDNA together. This finding suggests that the two morphs may live in contact with each other in the same body of groundwater and that they may be reproductively isolated species. Our results show that the eDNA approach is suitable and efficient in addressing questions in biogeography, evolution, taxonomy and conservation of the cryptic subterranean fauna.

The olm, *Proteus anguinus* Laurenti 1768, is a large amphibian endemic to subterranean waters of the Dinaric Karst, with a known range stretching between north-eastern Italy and southern Bosnia and Herzegovina. Despite the relatively broad expanse (60,000 km^2^) of karst topography in this region and decades of field surveys, *Proteus* has only been documented at around 300 sites^1^,^2^,^3^,^4^. These sites include caves where *Proteus* is recorded by visual observation or trapping, and springs where specimens may emerge during seasonal flooding^5^. While groundwater pollution and destruction of subterranean habitat are obvious threats to *Proteus*^6^,^7^, the negative anthropogenic impact cannot be determined without a reliable methodology to establish and monitor its presence.

Even with advances in speleobiology, progress in defining the true geographic distribution and diversity of its populations has been slow^8^. For example, despite much speculation, no physical evidence of the presence of *Proteus* in the Dinaric Karst of Montenegro has been documented. Furthermore, as recently as in 1986, a unique, darkly pigmented non-troglomorphic population of *Proteus* was discovered in south-eastern Slovenia^9^ and described as the subspecies *Proteus anguinus parkelj*^10^. The results of subsequent morphometric analyses^11^,^12^,^13^ supported its distinct taxonomic status, and phylogeographic analyses of mitochondrial DNA (mtDNA)^14^,^15^ confirmed the distinctiveness of its lineage, one, however, that is deeply nested in the *Proteus* phylogeny. The population of black *Proteus* has been documented at only four sites in an area of less than 2 km^2^ (refs 1, 7 and 16). In the same geographic region, but presumably in a different hydrogeological formation^17^,^18^, a closely related lineage^14^ of the troglomorphic, white *Proteus* subspecies (*Proteus anguinus anguinus*) has also been recorded at nine sites^1^,^9^ (also A. Hudoklin, pers. comm. 20 July 2015). If two such morphs co-existed in the same local habitat without hybridizing, they would likely be reproductively isolated from each other by an intrinsic barrier. However, this simple and powerful test of species status is rarely available in obligate subterranean organisms, because their habitat is patchy and their populations are usually fragmented and physically strongly isolated from each other^19^,^20^,^21^.

To detect species like *Proteus*, that are rare and difficult to observe with classical methods, detection of specific DNA released into the environment (environmental DNA or eDNA) is particularly useful^22^,^23^,^24^,^25^,^26^,^27^. The ubiquitous nature of DNA in aquatic environments and its rapid diffusion from its source means that in theory the presence of a specific animal can be detected anywhere within the water body and not just at its point of origin^28^,^29^. Furthermore, as DNA released into most environments becomes quickly degraded, the eDNA approach detects the recent presence of target species^30^ without the need for direct observation or trapping.

In this study we used *Proteus* individuals from the laboratory to develop a set of eDNA detection assays based on quantitative real-time polymerase chain reaction (qPCR) to examine the presence of *Proteus* in karst aquifers, where physical detection is difficult or impossible. First, we developed a SYBR green (Applied Biosystems) qPCR assay to search for *Proteus* eDNA in spring and cave water samples from the under-explored southernmost edge of the known range of *Proteus* in Herzegovina (southern Bosnia and Herzegovina) and outside of it in Montenegro. Second, we developed a TaqMan (Applied Biosystems) qPCR assay to discriminate the black *Proteus* eDNA from the white *Proteus* eDNA. Using this assay, we conducted a systematic inventory of *Proteus* in Bela Krajina (south-eastern Slovenia) to verify selected historic records of the white *Proteus*, to determine the maximum range of the black *Proteus* and to test for possible co-occurrence of the two morphs.

## Results

### *Proteus* eDNA detection by qPCR

**Figure 1:**
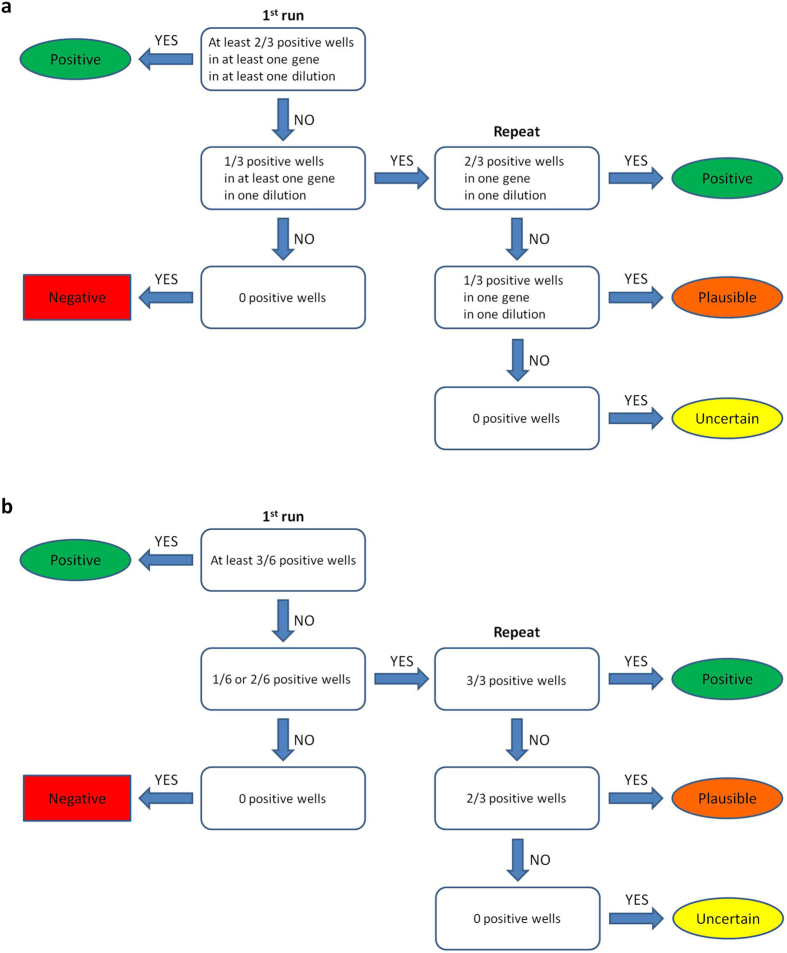
Evaluation of *Proteus* eDNA presence in a sample from the observed outcome of (**a**) SYBR qPCR assay and (**b**) TaqMan qPCR assay. Two mitochondrial DNA (mtDNA) regions (“genes”) were searched for in (**a**) and one mtDNA region was searched for in (**b**). The first run in both (**a**) and (**b**) included two concentrations (“dilutions”) of the template (data were pooled in **b**). All assays were performed in three parallel reactions (“wells”). See also the Methods section.

The sample validation procedure for the observed outcomes of qPCR tests is illustrated in Fig. 1. The Supplement lists the lower limit of detection and the confirmation of assay specificity for both SYBR and TaqMan qPCR assays. No false positives were observed.

Out of 23 sites in Herzegovina examined for eDNA by the SYBR qPCR assay, four were verified to harbour *Proteus* (confirmed through visual encounter by a reliable informant). Out of these, two scored positive and one plausible for its eDNA while the fourth was negative. In Bela Krajina (Slovenia), only one verified site was included in the analyses by the TaqMan qPCR assay and it scored positive. Out of additional four likely sites (within the known range of the black *Proteus*), three also were positive and one was negative.

In our estimations during sampling, the flow of the springs where *Proteus* eDNA was detected varied from springs and wells with discharge rates as low as 0.1 L/s to as high as 2000 L/s (see Supplementary Table S1). The maximum water temperature recorded during sampling was an exceptional 17 °C, with the median at 11.7 °C (see Supplementary Table S1). Once sampled, DNA in water degraded within several days when stored at 4 °C. By contrast, storage of dry filters at −20 °C sufficiently preserved the integrity of the DNA for at least two months, and storage of isolated DNA at −20 °C for at least four months.

### Detection of *Proteus* by eDNA in Herzegovina and Montenegro

**Figure 2:**
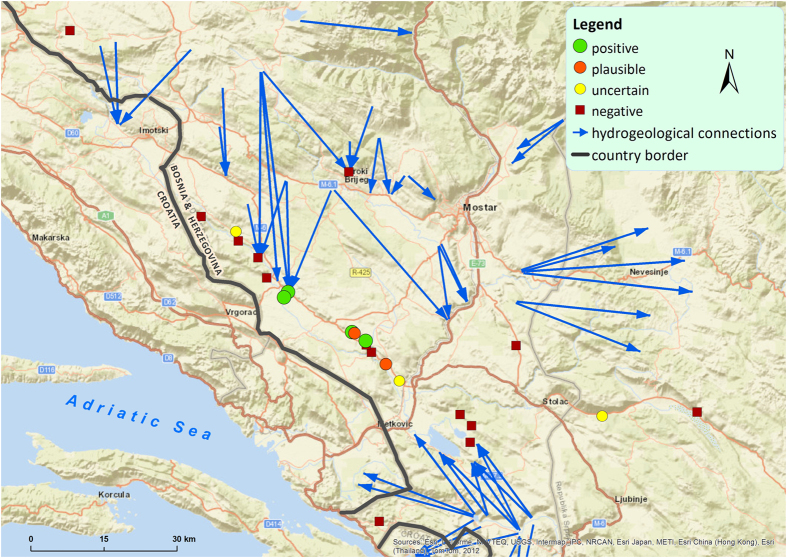
Map of sampling sites and results of eDNA analyses in Bosnia and Herzegovina. Categorisation of sites is explained in Fig. 1a and in the Methods section. The map was created using ArcGIS Desktop 10.3.1 (Esri 2015). Basemap used: World Street Map (Esri 2015). Source of hydrogeological data: http://diktas.iwlearn.org/im/hydrogeological-map-of-the-dinaric-karst (last accessed 5 October 2016).

In Herzegovina (Fig. 2), the springs Londža, Muša (no. 1 in Supplementary Table S1), Nezdravica (no. 14) and Izvor Bregave (no. 20) showed weak signals (one positive signal in three replicates) representing only one of the genes and were negative in the re-run. Hence, these samples are categorised as uncertain for the presence of *Proteus* eDNA (possibly at the limit of detection, although we cannot completely exclude contamination despite the precaution mechanisms). A private well in Gornji Trebižat (no. 2) and Vrelo Vakuf (no. 4) showed a weak signal (one positive signal in three replicates) representing only the mitochondrial control region, and this signal (one positive signal in three replicates) was again observed in the re-run. Therefore, these samples are categorised as plausible for containing *Proteus* eDNA. Here contamination is less likely as the signal was observed in independent runs. Perića Mlin (no. 3) and Kajtazovo Vrelo (no. 5) were positive for one gene, whereas the samples from Bunar kod Kuće Mehe Dizdarevića (no. 10) and Česma izpod Pogledovače (no. 11) were positive for both genes. Thus, these localities are interpreted as positive for the presence of *Proteus* eDNA.

**Figure 3:**
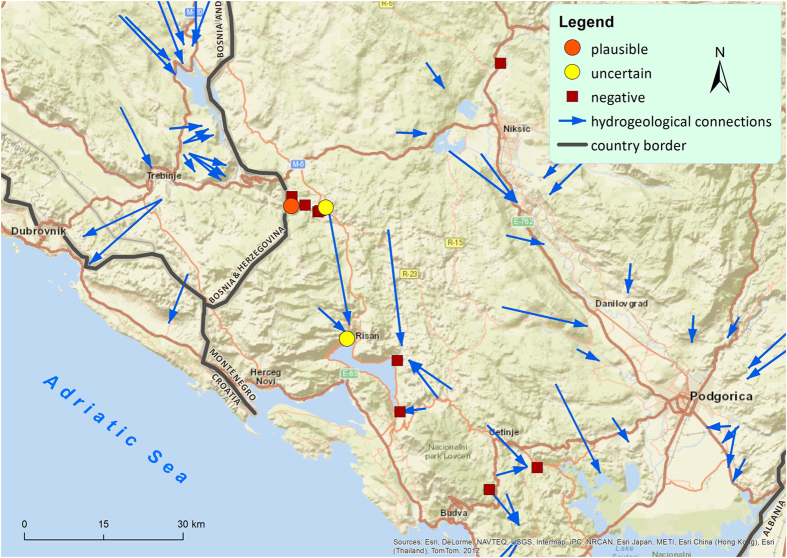
Map of sampling sites and results of eDNA analyses in Montenegro. Categorisation of sites is explained in Fig. 1a and in the Methods section. The map was created using ArcGIS Desktop 10.3.1 (Esri 2015). Basemap used: World Street Map (Esri 2015). Source of hydrogeological data: http://diktas.iwlearn.org/im/hydrogeological-map-of-the-dinaric-karst (last accessed 5 October 2016).

In Montenegro (Fig. 3), the cave Sopot (no. 24 in Supplementary Table S1) and the spring Izvor Grahovo 1 (no. 29) showed weak signals (one positive signal in three replicates) representing only one of the genes and were negative in the re-run. Therefore, these signals are interpreted as uncertain for the presence of *Proteus* eDNA. The spring Šanik (no. 33) also showed a weak signal representing only the 16S rRNA gene (one positive out of three replicates), but in this case the same signal was observed again in three separate re-runs. Therefore, this sample is interpreted as plausible to contain *Proteus* eDNA, and here contamination is less likely as the signal was observed in four independent runs.

### Detection of black and white *Proteus* by eDNA in Bela Krajina (Slovenia)

**Figure 4:**
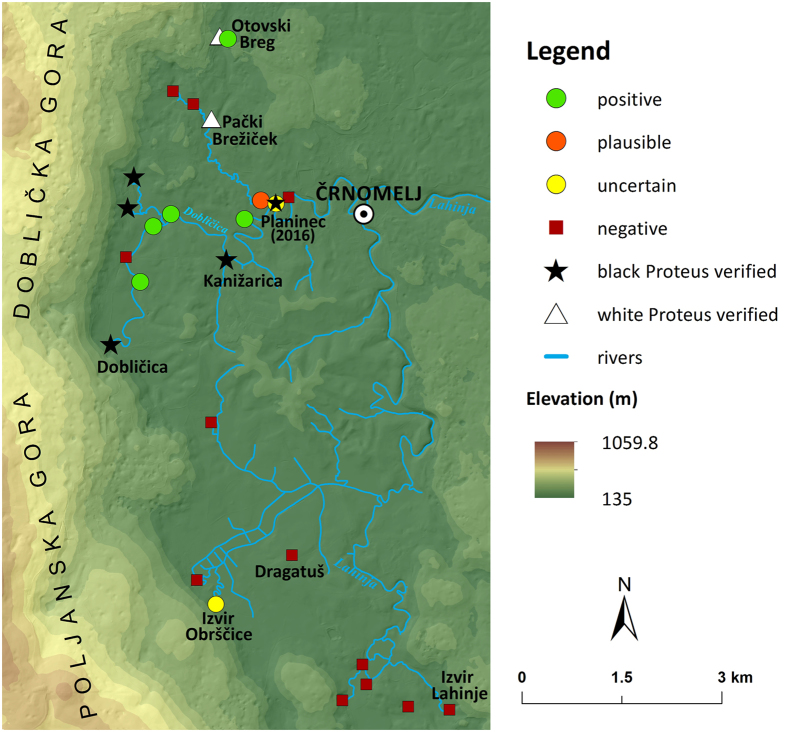
Map of sampling sites and results of eDNA analyses for *Proteus* in Bela Krajina (Slovenia). Circles and squares depict the results of eDNA analyses; stars and triangles represent known localities of the black or the white *Proteus*, respectively. Categorisation of sites is explained in Fig. 1b and in the Methods section. Doblička Gora and Poljanska Gora are the south-eastern foothills of the Kočevski Rog Plateau. The map was created using ArcGIS Desktop 10.3.1 (Esri 2015). Source of basemap: digital elevation model at 1:10,000 (http://www.e-prostor.gov.si/si/zbirke_prostorskih_podatkov/topografski_in_kartografski_podatki/digitalni_model_visin/digitalni_model_visin_5_x_5_m_dmv_5/, last accessed 5 October 2016).

*Proteus* eDNA was confirmed in six out of 19 samples analysed from Bela Krajina (Fig. 4). Except for Otovski Breg (no. 45 in Supplementary Table S1), a verified site with white *Proteus*, all of the positive sites were springs along the Dobličica River. Following the direction of the Dobličica River flow, there was a gradient of relative concentration of *Proteus* eDNA in these springs, as deduced from cycle threshold (Ct) values in combination with the fraction of positive replicates in each sample. In the south-to-north and west-to-east directions, these fractions were as follows: Izvir ob Dobličici BK D3 (no. 51) 6/6, Izvir ob Dobličici BK D4 (no. 52) 4/6, Izvir ob Izlivu Jelševnice v Dobličico BK A2 (no. 47) and Šprajcarjev Zdenec (no. 41) 6/6, Izvir v Svibniku (no. 54) 2/6 + 2/3, Planinec (no. 55) 1/6 + 0/3. Outside the immediate area of the Dobličica River, the Otovski Breg (no. 45) sample was positive in 5/6 reactions, and Izvir Obrščice (no. 40) in only 1/6 + 0/3 reactions. The latter and Planinec were not analysed further as the presence of *Proteus* eDNA in these springs was uncertain. All other analysed samples were negative for the presence of *Proteus* eDNA. Following our analyses, on the evening of November 1, 2016, a young black *Proteus* was observed by the last two co-authors in Planinec.

**Figure 5:**
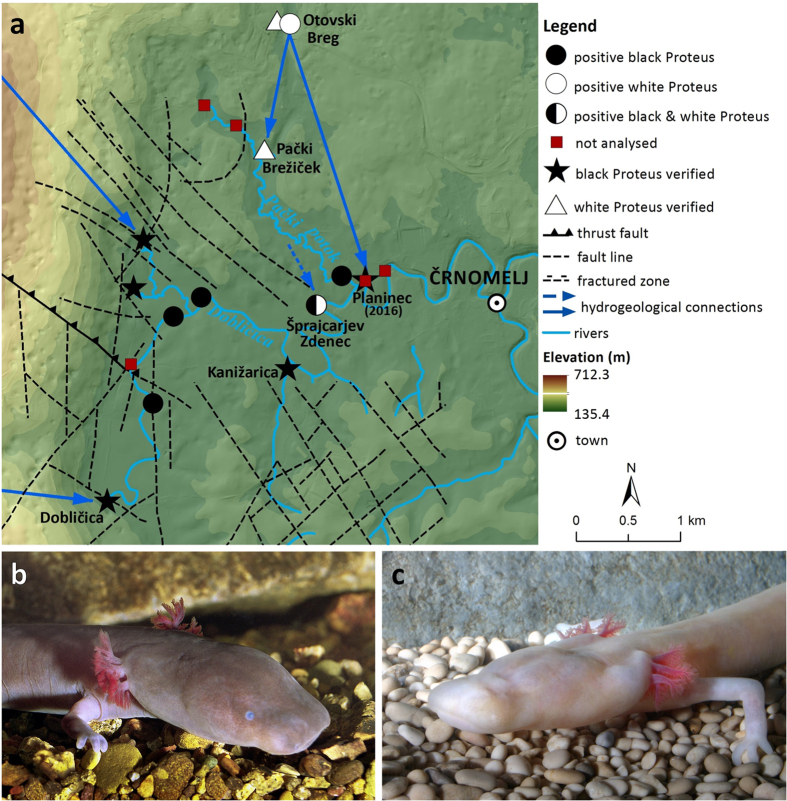
(**a**) Distribution of eDNA specific for (**b**) black and (**c**) white *Proteus* in spring samples in Bela Krajina (Slovenia). Circles and squares depict the results of eDNA analyses; stars and triangles represent known localities of the black or the white *Proteus*, respectively. The map was created using ArcGIS Desktop 10.3.1 (Esri 2015). Source of basemap: digital elevation model at 1:10,000 (http://www.e-prostor.gov.si/si/zbirke_prostorskih_podatkov/topografski_in_kartografski_podatki/digitalni_model_visin/digitalni_model_visin_5_x_5_m_dmv_5/, last accessed 5 October 2016). Sources of hydrogeological and geological layers: refs 17, 18 and 51.

Once *Proteus* eDNA was confirmed in a sample, it was tested further to determine if the eDNA belonged to the black or the white *Proteus* morph (Fig. 5). We thus detected black *Proteus* eDNA in five samples, all taken in springs along the Dobličica River, while the Otovski Breg (no. 45) sample was negative for black *Proteus* eDNA (0/6 reactions). The fraction of positive reactions again followed a northward and eastward gradient: Izvir ob Dobličici BK D3 (no. 51) 6/6, Izvir ob Dobličici BK D4 (no. 52) 3/6, Izvir ob Izlivu Jelševnice v Dobličico BK A2 (no. 47) 6/6, Šprajcarjev Zdenec (no. 41) 3/6, Izvir v Svibniku (no. 54) 2/6. We next analysed the same six samples for white *Proteus* eDNA. As expected, white *Proteus* eDNA was confirmed in the Otovski Breg sample (no. 45; 3/6), a known white *Proteus* site. Importantly, however, white *Proteus* eDNA was found in Šprajcarjev Zdenec (no. 41; 2/6 + 3/3), which was also positive for black *Proteus* eDNA. All other samples that were positive for black *Proteus* eDNA were negative for white *Proteus* eDNA. Sequencing of the PCR products amplified in the sample from Šprajcarjev Zdenec confirmed that both the black and the white haplotype were present in the sample.

## Discussion

Below we evaluate the basic parameters of our eDNA assay and its effectiveness in detecting *Proteus* in the laboratory as well as in different subterranean habitats.

A suitable eDNA assay for *Proteus* must be able to detect trace amounts of highly diluted DNA released by potentially very small populations. The qPCR technique is commonly used in achieving this goal^22^,^31^. When compared to classical PCR in combination with cloning and sequencing^27^, the real-time qPCR approach significantly improves the efficiency of eDNA detection and reduces the possibility of contamination as post-PCR analysis is omitted. Our tests showed that this method is suitable for detection of *Proteus* eDNA, including under the conditions encountered at karst springs.

A compromise between at least three factors is necessary for an optimal use of the eDNA assay for *Proteus*: (1) the time of sampling, (2) the amount of water filtered and (3) the lower detection limit of the method. The discharge from karst springs typically varies significantly through seasons in response to precipitation in the catchment area, with many springs inactive during the dry season^32^,^33^. As a consequence, sampling when water levels are optimal may be a challenge. When water levels are very high, eDNA may become too diluted or dispersed for detection. The latter may have been the case in a few springs in Herzegovina, which therefore required a repeated sampling to detect the presence of *Proteus* eDNA. Another concern when sampling for *Proteus* eDNA is collecting water samples without disturbing the sediment at very low water levels, while higher water levels may increase sediment transport through the karst aquifer. Even though sediment potentially holds more eDNA^34^,^35^, it also prevents efficient filtration. Furthermore, sediment can be a source of PCR inhibitors^36^,^37^. Monitoring PCR inhibition with the addition of synthetic DNA and corresponding primers and probes to each reaction, and diluting template DNA when inhibition is detected, is essential when analysing environmental samples for eDNA.

Exposed DNA in water gradually decays predominantly due to the effects of UV-light, heat and decomposition by microorganisms^31^,^38^,^39^. Because of the absence of light and due to the relatively low and constant temperatures of groundwater inhabited by *Proteus* (Supplementary Table S1; see also ref. 1), we expect eDNA to be relatively stable, which presumably facilitates detection. Furthermore, the eDNA fragments targeted in our assays are 100–150 bp long, i.e. short enough to persist in the environment^40^ despite degradation processes. As factors that affect DNA degradation may be less detrimental in subterranean streams, eDNA transport distance is likely increased in this habitat. Since eDNA transport in streams is greatly affected by discharge rates^41^,^42^, we timed the sampling, whenever and wherever it was possible, to the lowest water level and discharge rates that still allowed for efficient sampling.

The mitochondrial control region and flanking DNA and the 16S rRNA gene were chosen for our eDNA assay because a large set of sequences in *Proteus* is available for comparison and primer/probe design (see Supplementary Information). We believe this approach minimises the risk of not detecting a *Proteus* population due to an unknown variation in sequence. This especially applies to the primer set designed to bind to the conserved 16S rRNA gene, which appears to be general enough to be used in detection of any population within as well as outside the known range of *Proteus*. The primer pair designed to amplify a fragment of the control region, on the other hand, targets a more variable region of the mitochondrial genome. Therefore, the possibility that it could not bind efficiently to the hypothetical Montenegro population, which may be genetically distinct from all other known populations, cannot be excluded.

Compared to classical approaches of visual surveying or trapping, the eDNA analysis substantially improves our ability to detect *Proteus* in groundwater. This is most clearly observable in the results of the Bela Krajina survey, where the number of known sites with the black *Proteus* more than doubled after a single sampling. Under optimal water-level conditions, the sampling protocol developed here for *Proteus* eDNA is expected to yield at least a 75% detection rate at densities of at least one animal per 256 m^3^ of water (see Supplementary Information). Although the number of sites used to validate the detection probability and accuracy of the method was low, laboratory tests (see Supplementary Information) and hydrogeological data strongly support the results obtained for springs with hitherto unknown status. Similar detection probabilities were reported for epigean aquatic vertebrate species (e.g. refs 25, 42 and 43).

Testing the usefulness of the eDNA assay in field research in Herzegovina resulted in the detection of *Proteus* at locations where it has not been previously recorded. In the most recent list of localities in Bosnia and Herzegovina^3^, only seven out of a total of 57 are known in the greater Trebižat River area. We discovered four new *Proteus* localities by the eDNA method and two very likely to harbour *Proteus*. Following our analyses, the presence of *Proteus* was visually confirmed by cave diving at the spring Kajtazovo Vrelo (no. 5 in Supplementary Table S1; Z. Vlaho, pers. comm. 29 June 2016).

Here we also report the evidence for the existence of *Proteus* in Montenegro, based on a plausible sample of *Proteus* eDNA recorded at the spring Šanik (no. 33 in in Supplementary Table S1). Šanik is located 1.7 km from the nearest *Proteus* locality in Herzegovina (B. Lewarne, pers. comm. 22 June 2014). The proximity of the two sites suggests that they may be hydrogeologically connected and therefore may share a common *Proteus* population. Alternatively, they may depend on the same catchment area, which could include an upstream *Proteus* locality potentially serving only as a donor site for eDNA influx into Šanik. Since no hydrogeological surveys have been conducted in the region, neither a connection of Šanik to the locality in Herzegovina nor the location of its catchment area has been determined. Nonetheless, preliminary results suggest that the two cave systems could be hydrogeologically isolated from each other (B. Lewarne, pers. comm. 2 October 2016). Our results therefore favour the possibility that *Proteus* individuals are actually present at the sampled location and that the southern range of *Proteus* extends into the Dinaric Karst of Montenegro.

Next, using the eDNA assay, we report the discovery of new sites that may harbour the rare black *Proteus*, while its presence was visually confirmed in yet another one, which showed a faint trace of *Proteus* eDNA. Three of these springs lie outside the limits of its known range and represent an extension of its presumed range north-eastward, along with the general direction of the flow of the Dobličica River. The distance of the new easternmost site, the spring Planinec (no. 55 in Supplementary Table S1), from the nearest previously visually confirmed site, Kanižarica^16^, is 1.2 km (the actual extent of the cave system is unknown). Since we confirmed by visual observation that the occurrence of *Proteus* eDNA in a spring is indicative of its actual presence in close proximity, the present knowledge confines the black *Proteus* between the high plateau Kočevski Rog – which is lacking surface streams – in the west, along the Dobličica River to the confluence with the stream Pački Potok in the east.

As in most aquatic cave animals, ranges of *Proteus* populations are probably historically determined and highly restricted by the boundaries of present-day subterranean hydrogeological networks^15^. The white *Proteus* population appears to occupy a larger range, including sites in the Dolenjska region west of Kočevski Rog^14^. It should be noted, however, that while the maximum span of the black *Proteus* range to the east, north and south was established in the present study, the extent of its distribution to the west remains unknown. The observed descending gradient in concentration of eDNA following the Dobličica main stream flow appears to reflect a complex local network of underground connections that could receive inflow through both southwest-to-northeast and northwest-to southeast oriented faults as proven also by the water tracings made in the region^17^,^18^ (see Fig. 5). Therefore, the core of the black *Proteus’* distribution probably includes the contact zone between the Kočevski Rog Plateau and the Bela Krajina Plain as well as the south-eastern parts of Kočevski Rog. This conclusion is consistent with earlier predictions, when only one^9^ or two sites^10^ were known.

The discovery of the eDNA of both black and white *Proteus* syntopically in the spring Šprajcarjev Zdenec (no. 41 in Supplementary Table S1) represents direct evidence suggesting that these two populations may be in contact with each other. The finding is strongly supported by an existing (intermittent) hydrogeological connection^17^ with Otovski Breg, a nearby site occupied by the white *Proteus* (no. 45 in Supplementary Table S1). Because the relative concentration of eDNA of the white *Proteus* in Šprajcarjev Zdenec was similar to the relative concentration detected at Otovski Breg (sampled at around the same date), which cannot be explained by the difference in discharge rates of the two springs alone, we believe that it reflects the actual presence of the white *Proteus* in Šprajcarjev Zdenec. Passive eDNA dispersal would likely result in the presence of white *Proteus* eDNA in the spring closest to Šprajcarjev Zdenec (Izvir v Svibniku; no. 54 in Supplementary Table S1), irrespective of a later sampling date and alongside black *Proteus* eDNA found there. As our analysis showed the relative concentration of white *Proteus* eDNA in Šprajcarjev Zdenec to be similar to the relative concentration of black *Proteus* eDNA in this same spring, but at the same time we failed to detect white *Proteus* eDNA in nearby Izvir v Svibniku, we expect repeated additional sampling would support this conclusion.

Rare cases of subterranean syntopic occurrence of closely related lineages are valuable for the study of the poorly understood mechanisms of speciation and differentiation within the subterranean realm (e.g. ref. 44). The distribution of the black and white *Proteus* eDNA in Bela Krajina is in agreement with the existence of a potential reproductive barrier between these two lineages, at least regarding female mating preferences. Assuming gene flow between the two lineages, substantial introgression of the white *Proteus* mtDNA would predict the presence of white *Proteus* eDNA in the springs to the west of Šprajcarjev Zdenec, which was not detected. Similarly, if inter-lineage mating regularly occurred in the other direction, we would expect black *Proteus* eDNA to appear in the sample at Otovski Breg, a site which was found to harbour only white *Proteus* eDNA. Significantly, despite a low degree of sequence divergence between the two populations observed in the mitochondrial control region^14^,^15^,^45^, none of the comparative studies to date have detected any signs of their interbreeding, e.g. haplotype sharing^14^,^15^ or intermediate morphology^10^,^12^,^13^,^46^,^47^,^48^. In combination with these observations, eDNA data suggest that the two populations may represent independent species, but additional analyses are needed to resolve the taxonomic status of the present as well as of other apparently monophyletic groups of *Proteus*.

In conclusion, we have demonstrated that the qPCR-based eDNA method can be utilised for a rapid detection of a rare subterranean species inhabiting karst groundwater. Due to its high sensitivity (see Supplementary Information) and general applicability, the SYBR qPCR-based eDNA assay is appropriate for large-scale inventories of *Proteus* in groundwater throughout the Dinaric Karst, while the high specificity of the TaqMan qPCR-based eDNA assay makes this approach suitable for monitoring the distribution of closely related populations or taxa. Furthermore, as suggested by the results of the Bela Krajina survey in particular, the qPCR-based approach enables us to assess the relative abundance of *Proteus* eDNA in groundwater over a small spatial scale. Finally, we have shown that the eDNA approach can also be helpful in identifying potentially sympatric populations in the cryptic subterranean environment and therefore can be useful in the study of evolutionary history and taxonomy of subterranean taxa.

## Methods

### Study Design

Development of our methodology to detect traces of *Proteus* eDNA in water involved the following steps (see Supplementary Information): (1) development of specific oligonucleotides for eDNA detection with qPCR, (2) testing the specificity of the oligonucleotides on tissue samples, (3) testing the lower detection limit of the method in laboratory conditions and (4) testing the performance of the method in nature, at three verified sites in Slovenia (SYBR qPCR only). Two broad geographic regions in the south-eastern part of the Dinaric Karst were then investigated for the presence and distribution of *Proteus* using the SYBR qPCR assay, while the distribution of two morphs of *Proteus* in south-eastern Slovenia was surveyed using the TaqMan qPCR assay. Our eDNA methodology is in line with general recommendations for eDNA sampling, analysis and reporting^49^.

### Water filtration and eDNA extraction

At field sampling sites, 10 (exceptionally) to 20 L of water were collected taking care not to disturb the sediment during sampling. Samples were collected in brand-new 5- or 10-L plastic canisters and stored in a dark cool room until filtration.

Most samples were filtered within 24 hours after collection. Water samples were filtered through sterile 0.45 μm PES membrane filters (Sterlitech or Whatman) mounted on Nalgene polysulfone reusable bottle top filter holders (47 mm diameter), or through Nalgene MF75 series disposable bottle top filters with integral 0.45 μm SFCA membrane (Thermo Scientific) using a vacuum aspirator pump. Up to four filter membranes were used per sample, depending on the degree of clogging by sediment and other particles in the water. After filtration, the filters were rolled up using sterile disposable forceps, put into 5 ml tubes provided in the PowerWater DNA isolation kit (MoBio Laboratories/Qiagen) and stored at −20 °C until DNA extraction. DNA was extracted following the kit manufacturer’s instructions, except for a minor adjustment to concentrate the eluted DNA: the final elution volume was 50 μl for 20-L samples and 30 μl for 10-L samples.

### DNA amplification

#### SYBR chemistry eDNA assay

Two mtDNA regions (control region and 16S rRNA gene), were chosen to explore the presence of *Proteus* at the southernmost edge of its range (see Supplementary Information for details). A 106-bp fragment of the former and a 153-bp fragment of the latter were PCR-amplified using primer sets *PangCRF* (5′-GCGTTAATTACAAGGTGCACTTGG-3′), *PangCRR* (5′-TGTACCAGGTATTACCTTTAATGTTGG-3′), *Pang16SF* (5′-CTGCCTGCCCAGTGACAACA-3′) and *Pang16SR* (5′-CACGAGGAGATCAATTTCGCAGA-3′).

Before PCR amplification, distilled water dilutions of each eDNA sample were prepared in ratios of 1/4 and 1/16. A reaction mix of 15 μl total volume, which was applied to both target fragments, contained 7.5 μl of 2X SYBR Green Real-Time PCR Master Mix (Applied Biosystems), 0.15 μl of each of 100 μM primers, 1.2 μl of sterile PCR-grade water and 6 μl of eDNA sample. All DNA amplifications were performed on ViiA 7 Real-Time PCR System (Applied Biosystems) under the default thermo-cycling conditions for the Hold and Melt Curve Stages, while the PCR Stage involved 40 cycles with a 15-s denaturation step at 95 °C and a 45-s annealing step at 62.5 °C.

For each template dilution, both target fragments were amplified in triplicate using separate plates for each primer pair. A single 384-well qPCR plate contained between four to 12 samples, so that individual samples were separated by at least one empty row and column. In addition, each plate included six negative controls (double distilled and tap water from outside of *Proteus* range) and two positive controls (tissue DNA and eDNA extracted from the laboratory water tanks), for a comparison of melting curves.

Samples were scored positive for *Proteus* eDNA if at least two out of three replicate wells of at least one combination (dilution-primer pair) were positive by qPCR (see Fig. 1A). If only one of the three wells was positive by qPCR, the sample was re-analysed using the same primer combination. Again, if at least two of the three replicate wells were positive in the re-run, the sample was scored positive for *Proteus* eDNA. If only one of the three wells was positive in the re-run, the sample was considered plausible to contain *Proteus* eDNA. If all wells were negative in the re-run, the presence of *Proteus* eDNA in the sample was considered uncertain. Finally, samples with all negative wells in the first run were scored negative for *Proteus* eDNA and were not analysed further.

#### TaqMan chemistry eDNA assay

Three primer-probe combinations were designed to first test for the presence of *Proteus* eDNA in each sample and subsequently to determine whether the eDNA was characteristic for the black or the white *Proteus* (see Supplementary Information for details). To detect *Proteus* eDNA, we used the primer pair *Pa16SF* (5′-TACTGCCTGCCCAGTGACAA-3′) and *Pa16SR* (5′-TGCACGAGGAGATCAATTTCG-3′), which amplified 157 bp of the 16S rRNA gene, and a FAM-labelled TaqMan-MGB probe *PROTEUS* (5′-TTACGCTACCTTTGCACG-3′) that binds to *Proteus*-specific complementary region within the amplicon. To recognise the black *Proteus* eDNA, we used the primer pair *BPaCytbF* (5′-CATCCTACTGACATGGATCGGA-3′) and *BPaCytbR* (5′-GGCAGAGGTCTAGGAGTTTGTTTTC-3′), which amplified 146 bp of the mitochondrial cytochrome b (*cytb*) gene, and the TaqMan-MGB probe *BLACK* (5′-CATAATCCCATCAGCCGGA-3′). The probe and forward primer contained two black *Proteus*-specific nucleotides at positions 2 & 7, and 5 & 13, respectively. To recognise the white *Proteus* eDNA, we used the primer pair *WPaCytbF* (5′-CAGATGCCATCGTACTGACCTG-3′) and *WPaCytbR* (5′-TAGGAGTTTGTTTTCAGCCCATC-3′), which amplified 143 bp of the *cytb* sequence, and the TaqMan-MGB probe *WHITE* (5′-ATCGCCCTAATTCCATC-3′). The probe and forward primer contained two white *Proteus*-specific nucleotides at positions 7 & 12, and 12 & 20, respectively.

As all three probes were FAM-labelled, the three assays were performed in separate reactions. Each sample was tested undiluted and as a 1/4 dilution to minimise the effect of PCR inhibitors. Each probe-template combination was run in a triplicate. All reactions included a synthetic control DNA, corresponding primers and a VIC-labelled probe (all part of the TaqMan Mutation Detection IPC Reagent Kit, Applied Biosysytems) to either detect the presence of PCR inhibitors or confirm that the assay was carried out. Each 384-well test plate included at least three wells of negative control (double distilled water, assay-non-specific *Proteus* tissue DNA in concentration of 10 pg or higher) and a positive control (assay-specific *Proteus* DNA in concentrations of 10 and/or 100 pg or higher). Individual field samples were separated by at least two empty rows and columns. Samples that scored positive or plausible (see below) for *Proteus* eDNA were then tested for black and white *Proteus* eDNA on separate plates. For all samples and assays we used 10 μl reaction mixtures containing 5 μl of 2X TaqMan Environmental Master Mix 2.0 (Applied Biosystems), 0.5 μl of each of 10 μM primers, 0.5 μl of 2.5 μM FAM-labelled probe, 1 μl of 10X TaqMan Mutation Detection IPC Reagent (Applied Biosystems), 0.2 μl of 50X TaqMan Mutation Detection IPC Control DNA (Applied Biosystems) and 2.3 μl of sample. qPCR reactions were performed on the ViiA 7 System (Applied Biosystems) under default conditions (1 min annealing at 60 °C), except for an increase of the number of cycles to 60.

If a specific product was observed in at least three out of six reactions containing either undiluted or diluted sample, the sample was scored positive for the presence of respective eDNA (see Fig. 1B). On the other hand, samples with all negative wells were scored negative and were not analysed further. If, however, the specific product was observed in only one or two reactions with either undiluted or diluted sample, the assay was re-run in three replicates of 15 μl reactions with the same proportions of reagents as above and 3.5 μl of undiluted sample. If at least two out of three replicates were positive in the repeat, the presence of the respective eDNA marker in the sample was considered plausible. If none or one of the replicates were positive in the re-run, the presence of the respective eDNA marker in the sample was considered uncertain.

### Environmental DNA detectability assessment

The minimal density of *Proteus* in water at which its eDNA can still be detected (i.e. the lower detection limit of the SYBR and TaqMan qPCR assays) was determined from the animals hosted in controlled conditions as described in the Supplement. The approval for maintenance of live *Proteus* individuals was granted to the Laboratory by the Ministry of Environment and Spatial Planning of the Republic of Slovenia, Slovenian Environment Agency (Permit no. 35601-95/2009-4).

### Field survey

The authorities of all political entities visited were informed of the purpose of our fieldwork and granted its approval.

#### Trebižat River and Hutovo Blato areas in southern Bosnia and Herzegovina

Samples were collected in three rounds: March 31 – April 8, 2014, April 27 – May 10, 2014 and June 19–20, 2014. A total of 38 sites were visited (karst springs, caves and wells), of which 23 samples were analysed (see Supplementary Table S1). Because of sample transportation delays during the first field trip, several sites were visited twice.

#### Dinaric Karst in Montenegro

A wide area of the Dinaric Karst in Montenegro was sampled, including the Nikšić region, Skadar Lake region and the springs in Boka Kotorska Bay, Grahovo Polje and the territory of Banjan. Sampling in Montenegro was also organised in three rounds: October 3–4, 2013, November 16–22, 2013 and June 5–10, 2014. In total, 15 localities were visited, 11 of which were analysed (see Supplementary Table S1).

#### Bela Krajina, south-eastern Slovenia

Between July 20–29, 2015, 36 sites were visited, 13 of which were sampled (karst springs and a cave; see Supplementary Table S1). We also sampled tap water in Dragatuš, which is pumped from the groundwater source Dobličica, a known black *Proteus* site. After some precipitation, on November 2, 2015, five additional springs were sampled.

Sample collection. The sampling sites were selected on the basis of both published lists^1^,^3^ and unpublished sources of information on putative *Proteus* presence (reports in local media, interviews with local residents) as well as available information on hydrogeological connectivity to known localities^17^,^18^,^50^ (also http://diktas.iwlearn.org/im/hydrogeological-map-of-the-dinaric-karst) and, ultimately, hydrological conditions encountered during our visit. Samples were taken at low to medium water levels and during the lowest to average annual discharge rates of individual springs. Field samples were collected and filtered as randomly as possible, i.e. samples collected and filtered on the same day were never from adjacent sources.

The investigators performing the qPCR assays were blinded with respect to detailed hydrogeological information pertaining to individual samples. Rigorous controls for preventing and monitoring contamination were employed throughout the entire procedure (see Supplementary Information for details). The Supplement also provides the methods for GIS database construction and mapping of hydrogeological and geological data and information on *Proteus* sites.

## Additional Information

**Accession codes**: *Proteus* mitochondrial control region and rDNA sequences are deposited in GenBank, Accession Numbers KY523107–KY523177.

**How to cite this article:** Gorički, š. *et al*. Environmental DNA in subterranean biology: range extension and taxonomic implications for *Proteus. Sci. Rep.* **7**, 45054; doi: 10.1038/srep45054 (2017).

**Publisher's note:** Springer Nature remains neutral with regard to jurisdictional claims in published maps and institutional affiliations.

## Supplementary Material

Supplementary Information
