# Supplementary material for: Environmental DNA in subterranean biology: range extension and taxonomic implications for Proteus
Source: Sci Rep. 2017 Mar 27;7:45054. doi: 10.1038/srep45054 (PMC5366867; doi:10.1038/srep45054)
Supplement: Supplementary Information [file srep45054-s1.doc]

**Environmental DNA in subterranean biology: range extension and taxonomic implications for *Proteus***

**Špela Gorički^1^*†§, David Stanković^1,2,3^‡§, Aleš Snoj^2^, Matjaž Kuntner^4^, William R. Jeffery^5^, Peter Trontelj^6^, Miloš Pavićević^7^, Zlatko Grizelj^8^, Magdalena Năpăruş-Aljančič^1,9^, and Gregor Aljančič^1^**

^1^Society for Cave Biology, Tular Cave Laboratory, Oldhamska cesta 8a, 4000 Kranj, Slovenia.

^2^Department of Animal Sciences, Biotechnical Faculty, University of Ljubljana, Groblje 3, 1230 Domžale, Slovenia.

^3^Department of Life Sciences, University of Trieste, Via Licio Giorgieri 5, Trieste 34127, Italy.

^4^Institute of Biology, Scientific Research Centre, Slovenian Academy of Sciences and Arts, Novi trg 2, 1000 Ljubljana, Slovenia.

^5^Department of Biology, University of Maryland, College Park, MD 20742, USA.

^6^Department of Biology, Biotechnical Faculty, University of Ljubljana, Večna pot 111, 1000 Ljubljana, Slovenia.

^7^Biospeleological Society of Montenegro, Cara Lazarja 22, 81000 Podgorica, Montenegro.

^8^Scientific Research Society Versus, Vitina bb, 88326 Vitina, Bosnia and Herzegovina.

^9^University of Bucharest Research Institute, *ICUB*, Transdisciplinary Research Centre Landscape - Territory - Information Systems, CeLTIS, 91-93, Splaiul Independentei, 050095 Bucharest, Romania.

* [goricki.spela@gmail.com](mailto:goricki.spela@gmail.com)

† Corresponding address: Scriptorium biologorum – Biološka pisarna d.o.o., Nikola Tesla 6, 9000 Murska Sobota, Slovenia.

‡ Present address: Department of Life Sciences, University of Trieste, Via Licio Giorgieri 5, Trieste 34127, Italy.

§ These authors contributed equally to this work.

**Supplementary Information**

**Primer and probe design and selection**

Two mitochondrial DNA regions (16S rRNA gene and control region with flanking sequences), which have proven useful for species identification for numerous vertebrates (e.g. refs. 52–54) and phylogeographic studies of *Proteus*^14^ were chosen to explore the distribution of *Proteus* by eDNA detection. 16S rRNA gene and adjacent regions were amplified and sequenced using primers 12Sa (5'-AAAACIIGGATTAGATACCC-3'; modified after ref. 52) and 16Sbr (5'-CCGGTCTGAACTCAGATCA-3'; see ref. 55). Control region and flanking sequences were obtained as described in ref. 14.

**SYBR chemistry eDNA assay**

Several sets of primers specific to *Proteus* were designed based on the alignment of mitochondrial control region and flanking sequences of 172 individuals from 38 localities (GenBank accession numbers of 76 unique haplotypes: KY523107–KY523149 and DQ494754–DQ494786^14^) and of mitochondrial 16S rDNA sequences of 51 individuals from 32 localities (GenBank accession numbers of 28 unique haplotypes: KY523150–KY523177), constructed with the software MUSCLE^56^. Two primer pairs (see Materials and Methods) were selected for the SYBR qPCR assay due to their best performance in the control experiments. The performance of both primer pairs was confirmed against tissue samples of two genetic lineages of *Proteus* (black *Proteus* from Bela Krajina and white *Proteus* from Postojna-Planina cave system, both Slovenia). The specificity of primer pairs was further tested against rainbow trout (*Oncorhynchus mykiss*), great-crested newt (*Triturus carnifex*) and human DNA, and evaluated by melting curve analysis.

**TaqMan chemistry eDNA assay**

*Proteus*-specific primers that had already been tested as described above and used in the SYBR qPCR assay were adapted for use in the TaqMan qPCR assay and additional primers and probes were designed *de novo*. The alignments used for primer and probe design were the same as for the SYBR qPCR assay. Specifically, the alignment used to design lineage-specific probes and primers included 12 black *Proteus* individuals from Jelševnik in the Bela Krajina region, 15 white *Proteus* individuals belonging to its sister lineage and originating from nearby sites in Bela Krajina (Otovski Breg, Krupa, Stobe), further 25 white *Proteus* individuals of the same lineage but from sites in a more distant geographic area (Dolenjska) as well as individuals from other, genetically separated and geographically even more distant lineages (the Stična area, south-western Slovenia, Italy and the rest of *Proteus* range). All primers and probes were analysed using several software programs, including OligoAnalyzer (<https://eu.idtdna.com/calc/analyzer>), DNA Thermodynamics & Hybridization (<http://biophysics.idtdna.com/>) and Primer Express (Applied Byosystems). Their specificity was re-examined in a Genbank search (<http://www.ncbi.nlm.nih.gov/genbank/>) using the blastn algorithm for short sequences.

Performance of all primer-probe pairs was confirmed against tissue samples of three genetic lineages of *Proteus* (black *Proteus* from Bela Krajina, white *Proteus* from Bela Krajina and white *Proteus* from the Postojna-Planina Cave System, all Slovenia) and water samples from aquaria containing, respectively, black *Proteus* from Bela Krajina and white *Proteus* from the Postojna-Planina Cave System (live individuals of white *Proteus* from Bela Krajina were unavailable). The specificity of all combinations was further tested against rainbow trout (*Oncorhynchus mykiss*) or bullhead (*Cottus gobio*) DNA, and verified by sequencing products of selected positive reactions.

**Environmental DNA detectability assessment**

All experiments with live *Proteus* individuals hosted in the Tular Cave Laboratory respect all fundamental ethical principles and requirements valid in Slovenia, including those under the Charter of Fundamental Rights of the European Union. The approval for maintenance of live *Proteus* individuals was granted to the Laboratory by the Ministry of Environment and Spatial Planning of the Republic of Slovenia, Slovenian Environment Agency (Permit no. 35601-95/2009-4). The handling of live animals in the laboratory is supervised by a veterinarian.

**SYBR chemistry eDNA assay**

The minimal density of *Proteus* in water at which its eDNA can still be detected with the SYBR qPCR assay (i.e. the lower detection limit) was determined as follows (see Supplementary Fig. S1). A single adult specimen of white *Proteus* from the Postojna-Planina Cave System (approx. 230 mm total length) was placed into a new water tank holding 100 L of fresh tap water (no *Proteus* eDNA present). 10 L of water were sampled in weekly intervals for five weeks, replacing the water removed with fresh tap water. The water sample was subjected to a twofold serial dilution (1, 1/2, 1/4, 1/8, and 1/16) and 0.5 L of each dilution was filtered. Additional sampling and another serial dilution (1/16, 1/64, 1/128 and 1/256) was done after eight weeks. Environmental DNA attached to the filters was extracted as described in the Materials and Methods section. qPCR was run using 6 μl of template DNA in a 20-μl reaction mix with SYBR Green PCR Master Mix (Applied Biosystems) under the cycling conditions described in the Materials and Methods section. To avoid PCR inhibition, 1/5 and 1/25 dilutions of the template were used. Two independent qPCR replications were performed for each sample. Minimum detection limit of the method was calculated by extrapolation to a 10- or 20-L sample.

**TaqMan chemistry eDNA assay**

The lower detection limit of the TaqMan qPCR assay was determined similarly. One specimen of white *Proteus* from the Postojna-Planina Cave System (the same individual as used in the SYBR qPCR assay) and one specimen of black *Proteus* from Bela Krajina (approx. 300 mm total length) were put separately in clean 100-L glass water tanks filled with fresh tap water (no *Proteus* eDNA present). 10 L of water were removed in weekly intervals and replaced with fresh tap water. After six weeks, 10 L of water were sampled from both tanks and subjected to a twofold serial dilution (1, 1/2, 1/4, 1/16, 1/64, 1/256, 1/512 and 1/1024). 0.5 L of each dilution was filtered. Additional sampling and serial dilution (1, 1/2, 1/4, 1/8, 1/16, 1/32 and 1/64) was done after nine weeks. Environmental DNA attached to the filters was extracted as described in the Materials and Methods section. qPCR was run using 2.3 μl of undiluted template DNA in a 10-μl reaction mix as described in the Materials and Methods section. Three replicates were performed for each sample dilution. Minimum detection limit of the method was calculated by extrapolation to a 10- or 20-L sample. The lower detection limit was determined for probes “*ALL*” and “*BLACK*”; the probe “*WHITE*” could not be tested because a live white *Proteus* individual from Bela Krajina was unavailable.

**Field testing (SYBR chemistry eDNA assay)**

Before its application in the field survey, the performance of the SYBR qPCR assay was tested at three sites in Slovenia (see Supplementary Table S1) that were previously verified for the presence of *Proteus*^1^: Antonov Izvir (no. 35) and Virski Izvir (no. 36), which are karst springs, and Kompoljska Jama (no. 37), which is a cave locality. At each site a 10-L sample of water was collected. Tap water collected in Kranj and Ljubljana (Slovenia), obtained from the underlying aquifer, served as negative control. In parallel, 2–10 L of water were sampled from three permanent water tanks of known sizes hosting known numbers of *Proteus* in the Tular Cave Laboratory (Kranj, Slovenia) as a positive control.

**Contamination prevention**

Rigorous controls for preventing and monitoring contamination were employed throughout the entire procedure, including decontamination of workspace and re-usable equipment using commercial solutions, as well as chlorine-, autoclave- and UV-based sterilization. The sterilization procedure was modified from the standard protocol used in forensic medicine^57^ and from recommendations of the filter holder’s manufacturer (Thermo Scientific) as follows. Re-usable equipment was dismantled, scrubbed and left to soak for 20 min in a 5% solution of sodium hypochlorite (NaClO), followed by two washes in distilled water for 10 min each. After being sprinkled and incubated for 20 min with DNA-ExitusPlus solution (AppliChem), the equipment was rinsed again in distilled water for 5 min and when possible, exposed to UV irradiation for at least 20 min. Finally, all re-usable equipment was autoclaved for 20 min. To further prevent contamination, DNA extraction was conducted in a building where no extraction of DNA from *Proteus* tissue samples or post-PCR work is conducted. Additionally, DNA isolation, pre- and post-PCR analyses were each performed in a different room.

**GIS database and mapping**

Available hydrogelogical and geological data for the three countries^17,18,50^ (also <http://diktas.iwlearn.org/im/hydrogeological-map-of-the-dinaric-karst>, last accessed 5 October 2016,) and information on *Proteus* sites from published sources^1^ (also <http://www.geopedia.si/>, <http://www.natura2000.si/>, last accessed 5 October 2016) as well as from interviews during field work were georeferenced using ArcGIS 10.3.1. Desktop (Esri 2015) in two GIS databases: Herzegovina and Montenegro (41records) and Slovenia (44 records). The data collected during the survey include the following attributes: sampling status, eDNA results, locality type (*Proteus* presence), elevation and water temperature. Because of the large size of the area represented, the data gathered for Herzegovina and Montenegro were projected in WGS 1984 World Mercator coordinate system, using the D_ WGS _1984 datum and overlapped on World Street basemap (Esri 2015). For Slovenia, we used the accurate Gauss Kruger local coordinate system, with SI_D_48_Gauss Kruger datum. Furthermore, we processed the available digital elevation model at 1:10,000 (<http://www.e-prostor.gov.si/si/zbirke_prostorskih_podatkov/topografski_in_kartografski_podatki/digitalni_model_visin/digitalni_model_visin_5_x_5_m_dmv_5/>, last accessed 5 October 2016) to use as a basemap, in order to better interpret the karst surface in the Bela Krajina (Slovenia) region. The spatial scales used to map the distribution of *Proteus* range from 1: 670,000 (Montenegro) to 1: 40,000 (Slovenia).

**Supplementary Results**

All samples from controlled laboratory conditions with relatively high concentrations of *Proteus* eDNA were positive using the SYBR qPCR approach. Further, we confirmed the presence of *Proteus* at two field test sites in Slovenia (the spring Virski Izvir and the cave Kompoljska Jama; nos. 36 and 37 in Supplementary Table S1), while the third known locality of *Proteus* examined in Slovenia (the spring Antonov Izvir; no. 35) showed a plausible presence of *Proteus* eDNA. No false positives were detected.

**Environmental DNA detectability assessment**

Environmental DNA of *Proteus* in 100-L tanks was first detected by SYBR qPCR assay after two weeks, while the detection limit after eight weeks corresponded to the dilution of 1/128 (Supplementary Fig. S1). This coincides with one animal per 128 m^3^ of water when sampling 10 L, and 256 m^3^ of water when 20 L are sampled. When the test was conducted on the same animal using the TaqMan qPCR approach, the lower limit of detection for 20-L samples was one animal per 64 m^3^ of water (for both tested probes). We attribute this decrease in sensitivity to the smaller volume of template that could be used in the amplification reaction due to the higher complexity of the reaction mixture. The TaqMan qPCR tests also suggested that detection was dependent on the size of the animal: employing the same reaction mixture and template volume, the lower limit of detection increased to that observed in the SYBR qPCR assay (one animal per 256 m^3^ of water) when the larger animal was used.

**Assay specificity**

In the majority of SYBR qPCR reactions there was no DNA amplification when fish, newt or human DNA was used as template; if non-specific amplicons were present (primer dimers, homologous sequences), however, they could easily be distinguished from amplified *Proteus* DNA by the shape of melting curves. When non-*Proteus* DNA was used as template in TaqMan qPCR, no false positives were detected. However: when a tissue sample of black *Proteus* was analysed using the TaqMan qPCR assay for white *Proteus*, non-specific amplicons were present at template concentrations of 10 pg (in two reactions out of five) or higher (regularly). The non-specific product could easily be distinguished from the genuine one by (1) a significantly higher cycle threshold (Ct) value and (2) an obviously lower slope of the passive reference-normalised and baseline-adjusted reporter signal (ΔRn) curve plotted against cycle number, i.e. by a slower rate of amplification. Moreover, non-specific amplification was never observed in tests of aquarium water containing black *Proteus* eDNA. Direct sequencing of these products did not produce a discernible read. Because of all of the above, non-specific amplification was not considered likely to confound the interpretation of the results of field tests. We also emphasise that, due to homoplasies in DNA sequence, the TaqMan assays for the black and the white *Proteus* are not applicable outside the Bela Krajina and Dolenjska regions (south-eastern Slovenia).

**Supplementary References**

# 52. Kocher, T. D. *et al.* Dynamics of mitochondrial DNA evolution in animals: amplification and sequencing with conserved primers. *Proc. Natl. Acad. Sci. U.S.A.* **86**, 6196-6200 (1989).

# 53. Vences, M., Thomas, M., van der Meijden, A., Chiari, Y. & Vieites, D. R. Comparative performance of the 16S rRNA gene in DNA barcoding of amphibians, *Front. Zool.* **2**, 5, doi: 10.1186/1742-9994-2-5 (2005).

# 54. Yang, L. Species identification through mitochondrial rRNA genetic analysis. *Sci. Rep.***4**, 4089, doi: 10.1038/srep04089 (2014).

# 55. Simon, C., Franke, A. & Martin, A. The polymerase chain reaction: DNA extraction and amplification in *Molecular Techniques in Taxonomy* (eds Hewitt, G. M., Johnson, A. W. B. & Young, J. P. W.) 329-355 (Springer, Berlin, 1991).

# 56. Edgar, R. C. MUSCLE: multiple sequence alignment with high accuracy and high throughput. *Nucleic Acids Res.* **32**, 1792-1797 (2004).

# 57. Zupanič Pajnič, I. Extraction of DNA from human skeletal material in *Forensic DNA Typing Protocols* (ed. Goodwin, W.) 89-108 (Methods in Molecular Biology 1420, Springer Science&Business Media, New York, 2016).


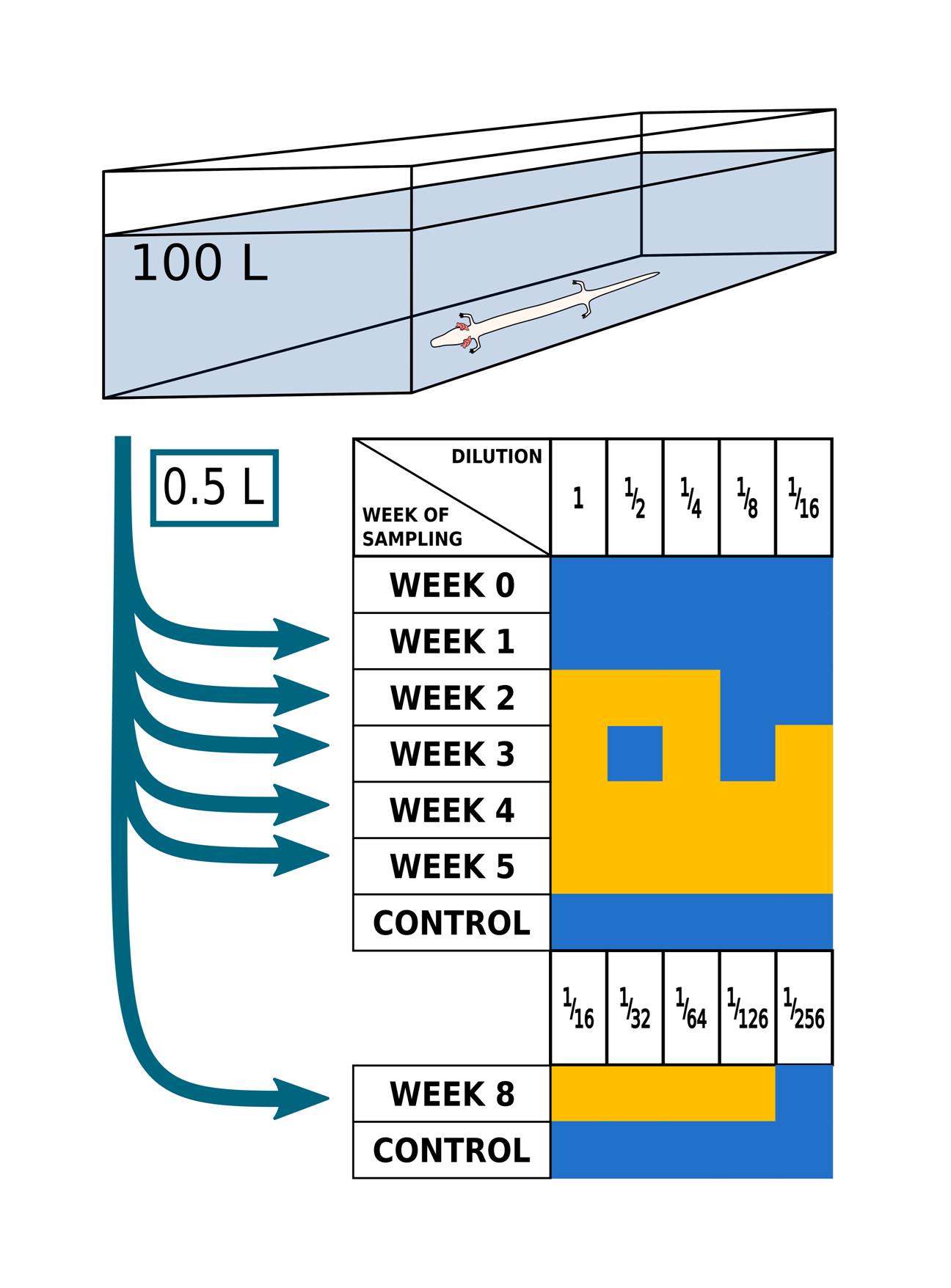


**Supplementary Figure S1. Schematic representation of the eDNA method calibration experiment.** Blue: negative for *Proteus* eDNA; yellow: positive for *Proteus* eDNA. A single *Proteus* individual was placed in a clean tank filled with tap water (Week 0). Water was sampled from the tank on weekly intervals and replaced with fresh tap water. Serial dilutions of the sample were made as shown and tested for *Proteus* eDNA. Minimum detection limit of the method was calculated by extrapolation to a 10- or 20-L sample.

**Supplementary Table S1.** **List of eDNA samplings and new records on *Proteus* presence.** Sites included in detection probability estimates are marked with * (SYBR chemistry) and **†** (TaqMan chemistry).

**I. BOSNIA AND HERZEGOVINA**

| **No.** | **Site (spring, cave or well)** | **Nearest settlement** | **GPS coordinates**  **(Lat. & Lon.)** | **Estimated**  **size** | **Water**  **temp. (°C)** | ***Proteus***  **presence** | **eDNA**  **data** | **Date of sampling** |
| --- | --- | --- | --- | --- | --- | --- | --- | --- |
| 1 | Londža, Muša | Muša, Čapljina | 43.116323  17.690188 | S | 12.6  / | P | -  ? | 4.4.2014  8.5.2014 |
| 2 | Private Well in Gornji Trebižat | Gornji Trebižat | 43.138552 17.665131 | S | 14.4  / | P | -  (+) | 4.4.2014  8.5.2014 |
| 3 | Cave at Perića Mlin | Studenci | 43.181603  17.603016 | S | 12  / | P | -  + | 2.4.2014  8.5.2014 |
| 4* | Vrelo Vakuf | Gornji Studenci | 43.18001  17.60728 | L | 12  / | V | -  (+) | 2.4.2014  8.5.2014 |
| 5* | Kajtazovo Vrelo | Donji Studenci | 43.169887 17.627965 | M | 12.4  / | V | -  + | 2.4.2014  8.5.2014 |
| 6 | Klokun | Klobuk | 43.281607 17.429480 | L | 12.4 | P | - | 6.4.2014 |
| 7 | Izvor Tihaljine | Peć Mlini | 43.336933 17.324700 | L | 11.7 | P | - | 6.4.2014 |
| 8 | Vrelo Studene / Sedra | Drače | 43.25454  17.44556 | M | / | P | - | 9.5.2014 |
| 9 | Božjak | Donji Studenci | 43.15461  17.63887 | M | / | P | - | 8.5.2014 |
| 10* | Bunar kod Kuće Mehe Dizdarevića | Vitina | 43.235705 17.484777 | S | / | V | + | 9.5.2014 |
| 11 | Česma izpod Pogledovače  [water pump] | Otok | 43.22818  17.47734 | / | / | V- | + | 9.5.2014 |
| 12 | Vrelo Modro Oko | Hržište | 43.30417  17.39308 | L | / | P | - | 9.5.2014 |
| 13* | Bunar kod Kuće Dragičević | Donji Studenci | 43.16437  17.62948 | S | /  / | V | -  - | 4.4.2014  7.5.2014 |

**Supplementary Table S1.** (Continued)

**I. BOSNIA AND HERZEGOVINA (continued)**

| **No.** | **Site (spring, cave or well)** | **Nearest settlement** | | **GPS coordinates**  **(Lat. & Lon.)** | | **Estimated**  **size** | **Water**  **temp. (°C)** | ***Proteus***  **presence** | **eDNA**  **data** | | **Date of sampling** | |
| --- | --- | --- | --- | --- | --- | --- | --- | --- | --- | --- | --- | --- |
| 14 | Nezdravica | Nezdravica | 43.31696  17.38867 | | M | | / | P | | ? | | 9.5.2014 |
| 15 | Londža, Derani | Derani | | 43.033385  17.821132 | | M | 12.5 | V- | - | | 3.4.2014 | |
| 16 | Jamica | Košćela | | 43.07066  17.80219 | | M | / | P | - | | 8.5.2014 | |
| 17 | Orah | Drijen | | 43.05565  17.82354 | | M | / | P | - | | 8.5.2014 | |
| 18 | Vrelo Lištice | Široki Brijeg | | 43.39662  17.59720 | | L | / | ? | - | | 9.5.2014 | |
| 19 | Idrizova Stublina | Trijebanj, Stolac | | 43.163687  17.905470 | | S | 10.6 | ? | - | | 5.4.2014 | |
| 20 | North-eastern Izvor Bregave | Predolje | | 43.069090 18.064154 | | L | 9.9 | ? | ? | | 5.4.2014 | |
| 21 | Vrijeka | Dabarsko polje | | 43.074016  18.239666 | | L | 10.3 | ? | - | | 5.4.2014 | |
| 22 | Blace | Neum | | 42.926783 17.653300 | | S | / | V- | - | | 8.4.2014 | |
| 23 | Bilobrkova Pećina | Vinica | | 43.58271  17.01599 | | S | / | ? | - | | 28.4.2014 | |

II. MONTENEGRO

| **No.** | **Site (spring, cave or well)** | **Nearest settlement** | **GPS coordinates**  **(Lat. & Lon.)** | **Estimated size** | **Water**  **temp. (°C)** | ***Proteus***  **presence** | **eDNA**  **data** | **Date of sampling** |
| --- | --- | --- | --- | --- | --- | --- | --- | --- |
| 24 | Sopot | Risan | 42.51363  18.68160 | L | / | ? | ? | 3.10.2013 |
| 25 | Izvor Ljute | Ljuta | 42.486417  18.767279 | L | / | ? | - | 3.10.2013 |
| 26 | Gurdič | Kotor | 42.4220862  18.7716838 | M | / | ? | - | 22.11.2013 |

**Supplementary Table S1.** (Continued)

**II. MONTENEGRO (continued)**

| **No.** | **Site (spring, cave or well)** | | **Nearest settlement** | | **GPS coordinates**  **(Lat. & Lon.)** | | **Estimated size** | | **Water**  **temp. (°C)** | | ***Proteus***  **presence** | | **eDNA**  **data** | | **Date of sampling** |
| --- | --- | --- | --- | --- | --- | --- | --- | --- | --- | --- | --- | --- | --- | --- | --- |
| 27 | Izvor Vrela | Vrela | | 42.324548 18.923711 | | S | | / | | ? | | - | | 22.11.2013 | |
| 28 | Obodska Pećina | Rijeka Crnojevića | | 42.352080  19.005075 | | M | | / | | ? | | - | | 16.11.2013 | |
| 29 | Izvor Grahovo 1 | Grahovo | | 42.673687 18.624793 | | S | | /  10.4 | | ? | | ?  - | | 21.11.2013  5.6.2014 | |
| 30 | Izvor Grahovo 2 | Grahovo | | 42.672515 18.621648 | | S | | / | | ? | | - | | 21.11.2013 | |
| 31 | Izvor Vidrovan | Vidrovan, Niksić | | 42.8574476 18.9426174 | | M | | / | | ? | | - | | 21.11.2013 | |
| 32 | Lower Spring of Kunska Rijeka | Nudo | | 42.68689  18.56252 | | S | | 11.3 | | ? | | - | | 10.6.2014 | |
| 33 | Šanik | Nudo | | 42.67494  18.56137 | | M | | 9.8 | | ? | | (+) | | 10.6.2014 | |
| 34 | Izvor Zaslapnice | Zaslap | | 42.680639 18.597627 | | S | | 8.9 | | ? | | - | | 10.6.2014 | |

**III. SLOVENIA**

**1. Field test sites (SYBR-chemistry)**

| **No.** | **Site (spring, cave or well)** | **Nearest settlement** | **GPS coordinates**  **(Lat. & Lon.)** | **Estimated size** | **Water**  **temp. (°C)** | ***Proteus***  **presence** | **eDNA**  **data** | **Date of sampling** |
| --- | --- | --- | --- | --- | --- | --- | --- | --- |
| 35 | Antonov Izvir | Mahniči | 45.779372  13.906529 | M | / | V | (+) | 21.7.2013 |
| 36 | Virski Izvir | Vir pri Stični | 45.948785  14.815247 | M | / | V | + | 17.11.2013 |
| 37 | Kompoljska Jama | Kompolje | 45.799583  14.731055 | M | / | V | + | 16.1.2014 |

**Supplementary Table S1.** (Continued)

**2. Bela krajina, SE Slovenia**

| **No.** | **Site (spring, cave or well)** | **Nearest settlement** | | **GPS coordinates**  **(Lat. & Lon.)** | | **Estimated size** | | **Water**  **temp. (°C)** | | ***Proteus***  **presence** | **eDNA**  **data** | **Date of sampling** |
| --- | --- | --- | --- | --- | --- | --- | --- | --- | --- | --- | --- | --- |
| 38 | Sihurna | | Talčji Vrh | | 45.5853195  15.1564501 | | S | | 12.5 | P | - | 20.7.2015 |
| 39 | Talački Breg | | Talčji Vrh | | 45.5835880  15.1603440 | | S | | - | P | - | 20.7.2015 |
| 40 | Izvir Obrščice | | Obrh pri Dragatušu | | 45.5161380  15.1473490 | | M | | 9.9 | ? | ? | 21.7.2015 |
| 41 | Šprajcarjev Zdenec | | Svibnik | | 45.5680490  15.1701580 | | S | | - | P | + | 22.7.2015 |
| 42 | Izvir Podturnščice | | Breznik | | 45.5194110  15.1608650 | | M | | 11.1 | ? | - | 22.7.2015 |
| 43 | Okno pri Nerajcu | | Mali Nerajec | | 45.5031766  15.1887176 | | M | | 11.2 | ? | - | 23.7.2015 |
| 44 | Izvir Lahinje | | Belčji Vrh | | 45.5018604  15.2092550 | | M | | 10.7 | ? | - | 24.7.2015 |
| 45**†** | Otovski Breg | | Otovec | | 45.5923493  15.1670193 | | M | | - | V | + | 25.7.2015 |
| 46 | Izvir Male Lahinje (Griči) | Mala Lahinja | | 45.5053093  15.1933205 | | M | | - | | ? | - | 25.7.2015 |
| 47**†** | Izvir ob Izlivu Jelševnice v Dobličico BK A2 | Jelševnik | | 45.5687570  15.1560300 | | S | | 17.0 | | P | + | 27.7.2015 |
| 48 | Vodovod OŠ Dragatuš [tap water] | Dragatuš | | 45.5227491  15.1791255 | | / | | 19.8 | | P | - | 27.7.2015 |
| 49 | Izvir Selskega Potoka | Kvasica | | 45.5406740  15.1636510 | | S | | - | | ? | - | 28.7.2015 |
| 50 | Jama Djud | Mala Lahinja | | 45.5023000  15.2013416 | | S | | - | | ? | - | 28.7.2015 |
| 51**†** | Izvir ob Dobličici BK D3 | Dobliče | | 45.5595960  15.1500810 | | S | | 13.3 | | P | + | 28.7.2015 |
| 52**†** | Izvir ob Dobličici BK D4 | Jelševnik | | 45.5671109  15.1526043 | | S | | 13.2 | | P | + | 2.11.2015 |

**Supplementary Table S1.** (Continued)

**2. Bela krajina, SE Slovenia (continued)**

| **No.** | **Site (spring, cave or well)** | **Nearest settlement** | **GPS coordinates**  **(Lat. & Lon.)** | **Estimated size** | **Water**  **temp. (°C)** | ***Proteus***  **presence** | **eDNA**  **data** | **Date of sampling** |
| --- | --- | --- | --- | --- | --- | --- | --- | --- |
| 53**†** | Vodovodni Jašek ob Mostu čez Dobličico BK D5  [services shaft filled with water] | Dobliče | 45.5629627  15.1473490 | S | 10.8 | P | - | 2.11.2015 |
| 54 | Izvir v Svibniku | Svibnik | 45.5705740  15.1732950 | S | 12.6 | P | (+) | 2.11.2015 |
| 55 | Planinec | Svibnik | 45.5703390  15.1761790 | S | 12.4 | V | ? | 2.11.2015 |
| 56 | Izvir ob Dobličici BK 52B | Svibnik | 45.5709740  15.1786340 | S | 11.8 | P | - | 2.11.2015 |

**Legend to Supplementary Table S1.**

| **Estimated size: est. discharge rate at time of sampling; classification after ref. 33.** | ***Proteus* presence** | **eDNA data** |
| --- | --- | --- |
| L large (over 100 L/s) | V verified (*Proteus* recently seen by a reliable informant or photographed) | + positive |
| M medium (10-100 L/s) | V- unverified (sightings by locals) | (+) plausible |
| S small (less than 10 L/s) | P plausible (adjacent to verified localities) | ? uncertain |
| / not applicable, no flow | ? unknown | - negative |
